# 2,3,4,5,6-Penta­fluoro-*trans*-cinnamic acid

**DOI:** 10.1107/S1600536813024513

**Published:** 2013-09-07

**Authors:** Angélica Navarrete, Ratnasamy Somanathan, Gerardo Aguirre

**Affiliations:** a Centro de Graduados e Investigación del Instituto Tecnológico de Tijuana, Apdo. Postal 1166, 22500, Tijuana, B.C., Mexico

## Abstract

The title compound, C_9_H_3_F_5_O_2_, crystallizes as O—H⋯O hydrogen-bonded carb­oxy­lic acid dimers that, together with C—H⋯F inter­actions and O⋯F [2.8065 (13) and 2.9628 (13) Å] and F⋯F [2.6665 (11), 2.7049 (12) and 2.7314 (12) Å] contacts, form a sheet-like structure. The sheets are stacked *via* short π–π inter­actions [centroid–centroid distance = 4.3198 (11) Å]. An intra­molecular C—H⋯F inter­action is also observed.

## Related literature

For related structures, see: Goud *et al.* (1995 ▶); Quan & Sun, (2013 ▶). For the biological activity of *N*-alkenyl amides, see: Brettle & Mosedale (1988 ▶). For fluorinated *N*-alkenyl amides, see: Aguirre *et al.* (1998 ▶).
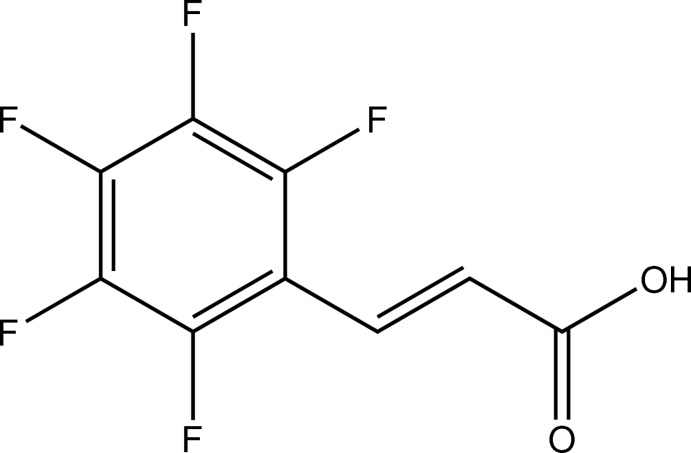


## Experimental

### 

#### Crystal data

C_9_H_3_F_5_O_2_
*M* *_r_* = 238.11
Triclinic, 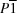

*a* = 4.3198 (9) Å
*b* = 7.4921 (17) Å
*c* = 13.225 (3) Å
α = 93.612 (12)°
β = 93.912 (12)°
γ = 103.769 (12)°
*V* = 413.37 (15) Å^3^
*Z* = 2
Mo *K*α radiation
μ = 0.21 mm^−1^
*T* = 100 K
0.35 × 0.30 × 0.09 mm

#### Data collection

Bruker APEXII CCD diffractometer
Absorption correction: multi-scan (*SADABS*; Bruker, 2008 ▶) *T*_min_ = 0.931, *T*_max_ = 0.982
11089 measured reflections
2405 independent reflections
2000 reflections with *I* > 2σ(*I*)
*R*_int_ = 0.026

#### Refinement

*R*[*F* ^2^ > 2σ(*F* ^2^)] = 0.031
*wR*(*F* ^2^) = 0.104
*S* = 0.70
2405 reflections
145 parameters
H-atom parameters constrained
Δρ_max_ = 0.50 e Å^−3^
Δρ_min_ = −0.23 e Å^−3^

### 

Data collection: *APEX2* (Bruker, 2008 ▶); cell refinement: *SAINT* (Bruker, 2008 ▶); data reduction: *SAINT*; program(s) used to solve structure: *SHELXS97* (Sheldrick, 2008 ▶); program(s) used to refine structure: *SHELXL97* (Sheldrick, 2008 ▶); molecular graphics: *SHELXTL* (Sheldrick, 2008 ▶); software used to prepare material for publication: *SHELXTL*.

## Supplementary Material

Crystal structure: contains datablock(s) I. DOI: 10.1107/S1600536813024513/gg2124sup1.cif

Structure factors: contains datablock(s) I. DOI: 10.1107/S1600536813024513/gg2124Isup2.hkl

Supplementary material file. DOI: 10.1107/S1600536813024513/gg2124Isup3.cml

Additional supplementary materials:  crystallographic information; 3D view; checkCIF report

## Figures and Tables

**Table 1:** Hydrogen-bond geometry (Å, °)

*D*—H⋯*A*	*D*—H	H⋯*A*	*D*⋯*A*	*D*—H⋯*A*
O2—H2*A*⋯O1^i^	0.84	1.81	2.6485 (13)	179
C7—H7⋯F4^ii^	0.95	2.47	3.4074 (14)	169
C8—H8⋯F5	0.95	2.22	2.8434 (14)	123

Symmetry codes: (i) 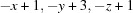; (ii) 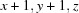.

**Fig. 1.**
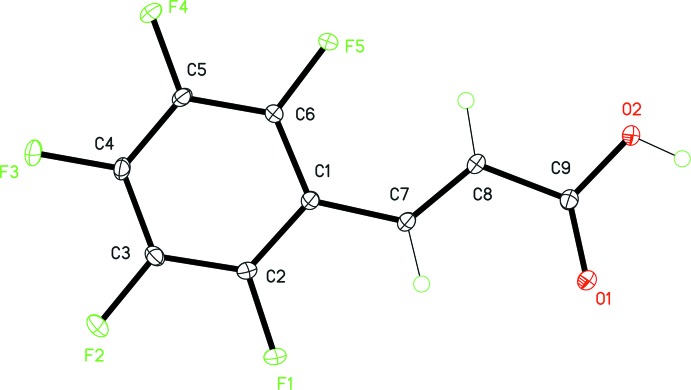
The molecular structure of the title compound with the atom numbering scheme, displacement ellipsoids are drawn at 30% probability level. H atoms are present as small spheres of arbitrary radius.

**Fig. 2.**
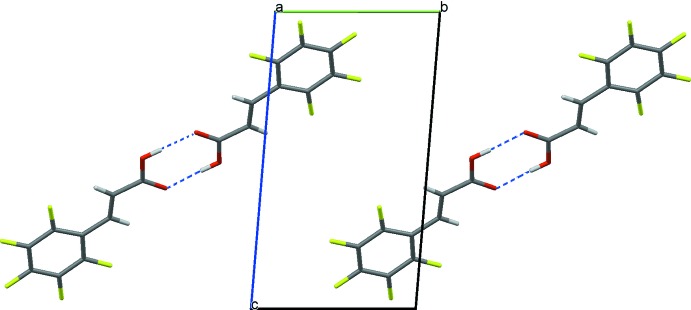
A packing diagram of (I). Intermolecular hydrogen bonds are shown as dashed lines.

## supplementary crystallographic information

### 1. Comment

N-Alkenyl amides are a rapidly emerging class of naturally occurring substances, widely distributed in higher plants, marine and microorganisms, and they exhibit an array of biological properties, including antibiotic, protein kinase inhibition an antitumor activity (Brettle & Mosedale, 1988). In particular we are interested in the synthesis of fluorinated N-alkenyl amides from commercially available fluorocinnamic acids and aldehydes (Aguirre *et al.*, 1998). In our synthesis of pentafluorinated enamide, we used 2,3,4,5,6-Pentafluoro-*trans*-cinnamic acid as the starting material, which is commercially available and herein we report the crystal structure (Fig. 1).

In the crystal structure adjacent networks are linked together *via* intermolecular hydrogen bond interactions (Table 1). The molecules form typical carboxylic acid dimers (Fig. 2) and stack *via*π–π interactions.

### 2. Experimental

2,3,4,5,6 Pentafluorocinnamic acid obtained from the Aldrich Chemical Company, was dissolved in chloroform. Slow evaporation at room temperature produces plates. One of which was cut provide the experimental sample. Melting point 154–156 °C.

^1^H NMR (CDCl_3_): δ 10.5 (s, 1H), 7.7 (d, J=16 Hz, 1H), 6.8 (d, J=16 Hz, 1H) p.p.m.; ^13^C NMR (CDCl_3_): δ 171.0 (*s*), 145.6 (d, J_C—F_=253 Hz), 142.1 (d, J_C—F_=253 Hz), 137.8 (d, J_C—F_=252 Hz), 130.6 (*s*), 125.3 (t, J_C—F_=5.8 Hz), 109.6 (d, J_C—F_=17 Hz) p.p.m.; ^19^F NMR (CDCl_3_): δ – 140.2 (dd, J=18,4 Hz), -151.3 (tt, J=20. 3 Hz), -162.4 (t,d, J=20,6 Hz) p.p.m.. EMIE m/e: [*M*]^+^ 238.

### 3. Refinement

Refinement for H atoms was carried out using a riding model, with distances constrained to: 0.98 Å for methine CH. Isotropic U parameters were fixed to *U*_iso_(H) = 1.2*U*_eq_(carrier atom) for aromatic CH.

### Crystal data

**Table:**

C_9_H_3_F_5_O_2_	*Z* = 2
*M**_r_* = 238.11	*F*(000) = 236
Triclinic, *P*1	*D*_x_ = 1.913 Mg m^−^^3^
Hall symbol: -P 1	Mo *K*α radiation, λ = 0.71073 Å
*a* = 4.3198 (9) Å	Cell parameters from 5831 reflections
*b* = 7.4921 (17) Å	θ = 3.1–30.6°
*c* = 13.225 (3) Å	µ = 0.21 mm^−^^1^
α = 93.612 (12)°	*T* = 100 K
β = 93.912 (12)°	Needle, colorless
γ = 103.769 (12)°	0.35 × 0.30 × 0.09 mm
*V* = 413.37 (15) Å^3^	

### Data collection

**Table:**

Bruker APEXII CCD diffractometer	2405 independent reflections
Radiation source: fine-focus sealed tube	2000 reflections with *I* > 2σ(*I*)
Graphite monochromator	*R*_int_ = 0.026
φ and ω scans	θ_max_ = 30.0°, θ_min_ = 1.6°
Absorption correction: multi-scan (*SADABS*; Bruker, 2008)	*h* = −6→6
*T*_min_ = 0.931, *T*_max_ = 0.982	*k* = −10→10
11089 measured reflections	*l* = −18→18

### Refinement

**Table:**

Refinement on *F*^2^	Primary atom site location: structure-invariant direct methods
Least-squares matrix: full	Secondary atom site location: difference Fourier map
*R*[*F*^2^ > 2σ(*F*^2^)] = 0.031	Hydrogen site location: inferred from neighbouring sites
*wR*(*F*^2^) = 0.104	H-atom parameters constrained
*S* = 0.70	*w* = 1/[σ^2^(*F*_o_^2^) + (0.091*P*)^2^ + 0.3271*P*] where *P* = (*F*_o_^2^ + 2*F*_c_^2^)/3
2405 reflections	(Δ/σ)_max_ = 0.001
145 parameters	Δρ_max_ = 0.50 e Å^−^^3^
0 restraints	Δρ_min_ = −0.23 e Å^−^^3^

### Special details

**Table:**

Geometry. All e.s.d.'s (except the e.s.d. in the dihedral angle between two l.s. planes) are estimated using the full covariance matrix. The cell e.s.d.'s are taken into account individually in the estimation of e.s.d.'s in distances, angles and torsion angles; correlations between e.s.d.'s in cell parameters are only used when they are defined by crystal symmetry. An approximate (isotropic) treatment of cell e.s.d.'s is used for estimating e.s.d.'s involving l.s. planes.
Refinement. Refinement of *F*^2^ against ALL reflections. The weighted *R*-factor *wR* and goodness of fit *S* are based on *F*^2^, conventional *R*-factors *R* are based on *F*, with *F* set to zero for negative *F*^2^. The threshold expression of *F*^2^ > σ(*F*^2^) is used only for calculating *R*-factors(gt) *etc*. and is not relevant to the choice of reflections for refinement. *R*-factors based on *F*^2^ are statistically about twice as large as those based on *F*, and *R*- factors based on ALL data will be even larger.

### Fractional atomic coordinates and isotropic or equivalent isotropic displacement parameters (Å^2^)

**Table:**

	*x*	*y*	*z*	*U*_iso_*/*U*_eq_	
C1	0.6800 (2)	0.93946 (14)	0.75051 (8)	0.0134 (2)	
C2	0.9181 (2)	0.96009 (14)	0.83065 (8)	0.0144 (2)	
C3	0.9520 (2)	0.81768 (15)	0.88834 (8)	0.0157 (2)	
C4	0.7427 (3)	0.64649 (14)	0.86844 (8)	0.0163 (2)	
C5	0.5033 (2)	0.62006 (14)	0.79029 (8)	0.0159 (2)	
C6	0.4761 (2)	0.76388 (14)	0.73261 (8)	0.0142 (2)	
C7	0.6612 (2)	1.09717 (14)	0.69294 (8)	0.0144 (2)	
H7	0.8193	1.2081	0.7125	0.017*	
C8	0.4473 (3)	1.10410 (14)	0.61580 (8)	0.0154 (2)	
H8	0.2826	0.9980	0.5926	0.018*	
C9	0.4709 (2)	1.27831 (14)	0.56750 (8)	0.0147 (2)	
F1	1.12678 (16)	1.12300 (9)	0.85325 (5)	0.01929 (16)	
F2	1.18772 (16)	0.84479 (10)	0.96299 (5)	0.02165 (17)	
F3	0.76994 (18)	0.50721 (10)	0.92323 (5)	0.02419 (18)	
F4	0.29659 (17)	0.45665 (9)	0.77171 (6)	0.02230 (17)	
F5	0.23906 (16)	0.72905 (9)	0.65828 (5)	0.01916 (16)	
O1	0.6780 (2)	1.41951 (11)	0.59342 (6)	0.01988 (18)	
O2	0.2455 (2)	1.26544 (11)	0.49321 (6)	0.01934 (18)	
H2A	0.2724	1.3661	0.4664	0.029*	

### Atomic displacement parameters (Å^2^)

**Table:**

	*U*^11^	*U*^22^	*U*^33^	*U*^12^	*U*^13^	*U*^23^
C1	0.0131 (4)	0.0122 (4)	0.0147 (4)	0.0025 (3)	0.0006 (3)	0.0021 (3)
C2	0.0135 (4)	0.0125 (4)	0.0157 (4)	0.0004 (3)	0.0002 (3)	0.0007 (4)
C3	0.0139 (4)	0.0190 (5)	0.0137 (4)	0.0040 (4)	−0.0018 (3)	0.0017 (4)
C4	0.0180 (5)	0.0144 (5)	0.0179 (5)	0.0052 (4)	0.0011 (4)	0.0067 (4)
C5	0.0155 (5)	0.0105 (4)	0.0204 (5)	0.0008 (4)	0.0005 (4)	0.0026 (4)
C6	0.0131 (4)	0.0136 (4)	0.0152 (4)	0.0022 (3)	−0.0016 (3)	0.0023 (3)
C7	0.0151 (4)	0.0109 (4)	0.0167 (4)	0.0019 (3)	0.0012 (4)	0.0028 (3)
C8	0.0168 (4)	0.0113 (4)	0.0175 (5)	0.0022 (3)	0.0006 (4)	0.0032 (3)
C9	0.0161 (4)	0.0129 (4)	0.0157 (4)	0.0043 (4)	0.0012 (3)	0.0023 (3)
F1	0.0182 (3)	0.0145 (3)	0.0207 (3)	−0.0033 (2)	−0.0033 (2)	0.0010 (2)
F2	0.0195 (3)	0.0258 (4)	0.0182 (3)	0.0046 (3)	−0.0065 (3)	0.0033 (3)
F3	0.0281 (4)	0.0184 (3)	0.0267 (4)	0.0059 (3)	−0.0029 (3)	0.0122 (3)
F4	0.0220 (3)	0.0117 (3)	0.0294 (4)	−0.0028 (2)	−0.0029 (3)	0.0058 (3)
F5	0.0165 (3)	0.0153 (3)	0.0223 (3)	−0.0008 (2)	−0.0077 (2)	0.0037 (2)
O1	0.0215 (4)	0.0132 (4)	0.0223 (4)	0.0002 (3)	−0.0048 (3)	0.0050 (3)
O2	0.0205 (4)	0.0137 (4)	0.0220 (4)	0.0017 (3)	−0.0059 (3)	0.0056 (3)

### Geometric parameters (Å, º)

**Table:**

C1—C2	1.4000 (14)	C5—C6	1.3814 (14)
C1—C6	1.3941 (14)	C6—F5	1.3363 (12)
C1—C7	1.4606 (14)	C7—C8	1.3408 (15)
C2—F1	1.3360 (12)	C7—H7	0.9500
C2—C3	1.3797 (15)	C8—C9	1.4740 (15)
C3—F2	1.3388 (12)	C8—H8	0.9500
C3—C4	1.3800 (15)	C9—O1	1.2244 (13)
C4—F3	1.3304 (12)	C9—O2	1.3172 (13)
C4—C5	1.3814 (15)	O2—H2A	0.8400
C5—F4	1.3298 (12)		
			
C6—C1—C2	115.35 (9)	C4—C5—C6	120.03 (10)
C6—C1—C7	125.16 (9)	F5—C6—C5	116.98 (9)
C2—C1—C7	119.49 (9)	F5—C6—C1	120.37 (9)
F1—C2—C3	117.27 (9)	C5—C6—C1	122.65 (9)
F1—C2—C1	119.82 (9)	C8—C7—C1	127.75 (10)
C3—C2—C1	122.91 (10)	C8—C7—H7	116.1
F2—C3—C4	120.01 (10)	C1—C7—H7	116.1
F2—C3—C2	120.26 (10)	C7—C8—C9	119.20 (10)
C4—C3—C2	119.73 (10)	C7—C8—H8	120.4
F3—C4—C3	120.83 (10)	C9—C8—H8	120.4
F3—C4—C5	119.85 (10)	O1—C9—O2	123.66 (10)
C3—C4—C5	119.32 (9)	O1—C9—C8	123.65 (10)
F4—C5—C4	119.82 (9)	O2—C9—C8	112.69 (9)
F4—C5—C6	120.15 (9)	C9—O2—H2A	109.5
			
C6—C1—C2—F1	179.53 (9)	C3—C4—C5—C6	0.34 (16)
C7—C1—C2—F1	−0.46 (15)	F4—C5—C6—F5	−0.89 (15)
C6—C1—C2—C3	0.18 (16)	C4—C5—C6—F5	−179.94 (9)
C7—C1—C2—C3	−179.81 (10)	F4—C5—C6—C1	178.10 (9)
F1—C2—C3—F2	−0.48 (15)	C4—C5—C6—C1	−0.94 (17)
C1—C2—C3—F2	178.88 (9)	C2—C1—C6—F5	179.63 (9)
F1—C2—C3—C4	179.89 (9)	C7—C1—C6—F5	−0.38 (16)
C1—C2—C3—C4	−0.74 (17)	C2—C1—C6—C5	0.67 (16)
F2—C3—C4—F3	0.59 (16)	C7—C1—C6—C5	−179.34 (10)
C2—C3—C4—F3	−179.78 (9)	C6—C1—C7—C8	1.45 (18)
F2—C3—C4—C5	−179.16 (9)	C2—C1—C7—C8	−178.56 (10)
C2—C3—C4—C5	0.47 (16)	C1—C7—C8—C9	−179.90 (10)
F3—C4—C5—F4	1.54 (16)	C7—C8—C9—O1	0.40 (17)
C3—C4—C5—F4	−178.71 (9)	C7—C8—C9—O2	−179.81 (9)
F3—C4—C5—C6	−179.41 (9)		

### Hydrogen-bond geometry (Å, º)

**Table:**

*D*—H···*A*	*D*—H	H···*A*	*D*···*A*	*D*—H···*A*
O2—H2*A*···O1^i^	0.84	1.81	2.6485 (13)	179
C7—H7···F4^ii^	0.95	2.47	3.4074 (14)	169
C8—H8···F5	0.95	2.22	2.8434 (14)	123

Symmetry codes: (i) −*x*+1, −*y*+3, −*z*+1; (ii) *x*+1, *y*+1, *z*.
